# Impact of homologous recombination on core genome phylogenies

**DOI:** 10.1186/s12864-020-07262-x

**Published:** 2020-11-25

**Authors:** Caroline M. Stott, Louis-Marie Bobay

**Affiliations:** Department of Biology, University of North Carolina Greensboro, 321 McIver Street, PO Box 26170, Greensboro, NC 27402 USA

**Keywords:** Phylogeny, Recombination, Prokaryotes, Core genome

## Abstract

**Background:**

Core genome phylogenies are widely used to build the evolutionary history of individual prokaryote species. By using hundreds or thousands of shared genes, these approaches are the gold standard to reconstruct the relationships of large sets of strains. However, there is growing evidence that bacterial strains exchange DNA through homologous recombination at rates that vary widely across prokaryote species, indicating that core genome phylogenies might not be able to reconstruct true phylogenies when recombination rate is high. Few attempts have been made to evaluate the robustness of core genome phylogenies to recombination, but some analyses suggest that reconstructed trees are not always accurate.

**Results:**

In this study, we tested the robustness of core genome phylogenies to various levels of recombination rates. By analyzing simulated and empirical data, we observed that core genome phylogenies are relatively robust to recombination rates; nevertheless, our results suggest that many reconstructed trees are not completely accurate even when bootstrap supports are high. We found that some core genome phylogenies are highly robust to recombination whereas others are strongly impacted by it, and we identified that the robustness of core genome phylogenies to recombination is highly linked to the levels of selective pressures acting on a species. Stronger selective pressures lead to less accurate tree reconstructions, presumably because selective pressures more strongly bias the routes of DNA transfers, thereby causing phylogenetic artifacts.

**Conclusions:**

Overall, these results have important implications for the application of core genome phylogenies in prokaryotes.

**Supplementary Information:**

The online version contains supplementary material available at 10.1186/s12864-020-07262-x.

## Background

Phylogenetic approaches have been developed to reconstruct relationships between species and, in sexual organisms, these methods are not applicable to reconstruct relationships between individuals due to the exchange of large chromosomal fragments by crossovers at each generation. In contrast, microbial organisms are technically asexual and the lack of meiosis at each generation opens the possibility to reconstruct the phylogenetic tree of sets of related strains. These approaches have been widely applied to microbial organisms and the entire core genome (i.e. the set of genes shared by all the strains of the species) is usually used to reconstruct the tree of intraspecies relationships. Multiple artifacts can be encountered when constructing phylogenetic trees as exemplified by the frequent incongruence between gene trees and species trees [1–6], and the source of these incongruencies can be both methodological and biological [7–9].

Several studies have shown that by using the entire set of core genes, it is possible to obtain robust and consistent trees [10–12] for individual prokaryotic species. However, more and more studies are pointing out the pervasive role of homologous recombination in prokaryotes [13, 14] and some methods are now attempting to reconstruct intraspecies trees while accounting for recombination events [15–17]. Although originally perceived as purely clonal, it appears that, in fact, most prokaryotes engage in recombination at various levels ranging from clonal (i.e. complete absence of recombination) to panmictic species (i.e. high recombination rates approaching the ones of sexual organisms) [13, 14, 18, 19]. Multiple works have found evidence that recombination can significantly impact tree reconstruction [20–30]. However, very few studies have investigated this question in the context of whole-genome phylogeny [31, 32] and prokaryote phylogeny [33]. Therefore, it remains unclear to what extent homologous recombination impacts the ability of phylogenetic methods to reconstruct true phylogenetic trees of prokaryote species [34]. In theory, species experiencing little or no recombination should be less impacted by conflicting phylogenetic signals and reliable phylogenetic trees are expected to be obtained for these species. At the other end of the spectrum, prokaryotic species exchanging DNA at high rate are expected to yield poorly resolved trees, but the amount of recombination that a core genome can withstand while preserving true phylogenetic signal has not been investigated in depth.

Recently, a study has shown that almost all individual sites with phylogenetic signal were in disagreement with the core genome phylogeny of *Escherichia coli* [35]. The authors concluded that recombination was responsible for these incongruencies and that the resulting tree topology was robust, reproducible, but artifactual. These conclusions and other studies [18, 36, 37] have important ramifications for the application of core genome phylogenies which are widely applied to prokaryotes. However, two questions remain unanswered: i) what amount of recombination a given core genome can withstand without yielding artifactual trees? ii) what factors contribute to the robustness or sensitivity of phylogenetic trees to recombination?

In this study, we demonstrate that even when the near totality of individual sites of the core genome are incongruent with the core genome phylogeny, the true topology can still be retrieved from the dataset. This surprising result can be explained by the fact that individual sites that are incongruent with the topology can still retain some of the true phylogenetic signal. Therefore, the cumulation of all sites into one large alignment can offset the incongruent signal of individual sites. We further use in silico simulations and empirical core genome datasets to estimate to what extent homologous recombination impacts core genome phylogenies. Our analyses indicate that, usually, recombination rates do not completely disrupt the phylogenetic signal. However, recombination does impact tree topologies, suggesting that many phylogenetic trees reconstructed from core genome sequences are not thoroughly accurate. Finally, we identify that core genome trees are typically less reliable when genome-wide selective pressures are high, whereas core genome trees built for species evolving under more relaxed selection are much more robust to recombination. These results suggest that the combined effect of recombination and selection is affecting the reconstruction of core genome phylogenies in prokaryotes.

## Results and discussion

### True phylogenies can be inferred from incongruent sites

Several studies have demonstrated that gene trees are often incongruent with core genome phylogenies, opening the possibility that core genome phylogenies might correspond to artifacts [18, 36, 37]. It has been observed that most sites in the core genome can be incongruent with the overall tree topology and this has been considered as evidence that the reconstructed trees are not representative of the true evolutionary history of the strains [35]. We first tested whether true phylogenetic trees can be recovered when nearly all sites of the core genome are inconsistent with the overall tree topology. As a proof-of-concept we evolved a 2 Mb-long core genome in silico following a branching process. In order to obtain a realistic tree and core genome, we used the phylogenetic tree generated on a real dataset previously published [38]. We chose the tree and the parameters of the core genome of *Acinetobacter pittii* to conduct the simulations because the average bootstrap supports of this tree were closest to the average bootstrap support of our set of trees (average bootstrap support of 89%, see below). The real tree topology and branch lengths were used to simulate the evolution of the core genome evolving clonally (i.e. only by mutations). As expected, building the phylogenetic tree on the simulated dataset results in the same tree that was used to simulate the evolution of the core genome. Since the simulations were run from a real dataset, two nodes were poorly resolved in the simulated tree (these nodes were also poorly resolved in the real tree).

**Fig. 1:**
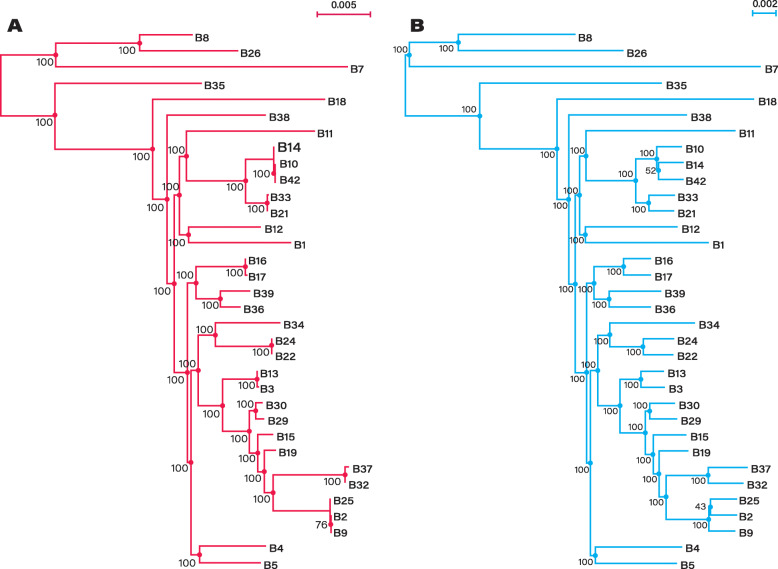
Robustness of tree topology to recombination. **a** Phylogenetic tree built on the clonal simulation of the core genome of *A. pittii*. The tree was evolved in silico without recombination. **b** Phylogenetic tree obtained from the simulated core genome of *A. pittii* recombined in silico. Each site of the clonal core genome with phylogenetic signal was exposed to exactly one recombination event. As a result, nearly all informative sites (96.4%) are incongruent with the true topology of the tree

We then used the core genome of the clonal simulation to generate a new core genome alignment while introducing exactly one random recombination event at each polymorphic site with phylogenetic signal (i.e. all sites with two or more alleles, except singletons). For instance, for a site composed of two alleles A and C (each present in at least two strains), we randomly picked one of these strains and re-affected the other allele (a C instead of an A or the opposite). Our procedure resulted in exactly one recombination event at each site containing phylogenetic signal and, as expected, the vast majority of alleles (96.4%) became incongruent with the phylogeny obtained from the clonal simulation (the true phylogeny). Note that, by chance, a few recombinant alleles were still congruent with the true phylogeny when the allele was transferred to a sister strain. We then built the phylogenetic tree from this recombined alignment and, despite the fact that only 3.6% of informative alleles support the true phylogeny, we obtained the same tree topology as the real tree (Fig. 1). A single node was incongruent in the two trees, but this node was one of the two unresolved nodes in the clonal and the true phylogeny. These results indicate that even when nearly all informative alleles are incongruent with the true phylogeny, it is still possible to retrieve the true phylogeny of the core genome. It is frequently observed that most gene trees are incongruent with the core genome phylogeny [39], but it does not necessarily imply that the true phylogeny cannot be recovered. Although this result might seem counterintuitive, it can be attributed to the fact that the simulated recombination events were random and only affected two strains at a time. As a consequence, each site still retains most of the phylogenetic signal, i.e. the strains grouped together by each site of the core genome were indeed more related to each other except for the one strain that was re-assigned. Combining the signal of all sites together allowed for the retrieval of the true phylogenetic signal of the core genome. This result demonstrates that it is theoretically possible to recover correct phylogenetic signals even when all sites are incongruent with the true phylogeny of the core genome.

### Impact of recombination rate on tree inference

**Fig. 2:**
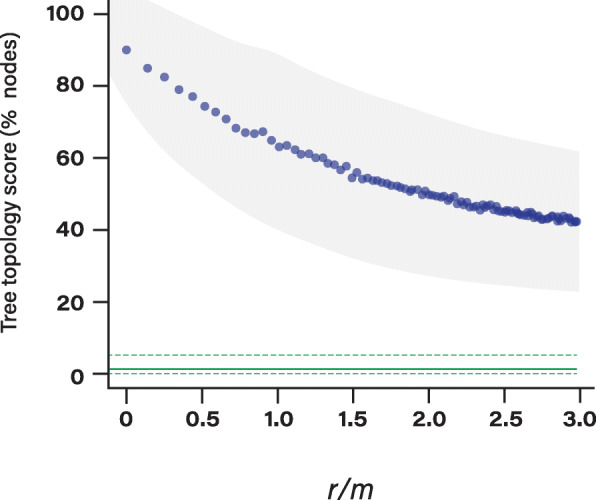
Impact of recombination rate (*r/m*) on tree inference. We evolved the core genome of 100 species with various levels of recombination rates. The tree topology score (TTS) represents the % of identical nodes between the simulated trees and the real trees. Reported TTS and *r/m* values on the graph represent the average values computed across the set of 100 species. Grey areas represent the standard deviation across the 100 species. Horizontal green lines represent the average (plain line) percent and standard deviation (dashed lines) of nodes expected to be identical if the trees were random

The previous result indicates that correct phylogenetic trees can be built from core genomes even though virtually every informative site can be incongruent with the true phylogeny. However, these simulations were conducted by introducing exactly one recombination event at each informative site and it is difficult to compare this analysis to the actual levels of recombination occurring across prokaryote species. To assess the effect of recombination rate on our ability to reconstruct accurate phylogenies in prokaryotes, we simulated the evolution of core genomes with various levels of recombination using *CoreSimul* [40]. In order to mimic realistic conditions, we used a previously published set of 100 core genomes encompassing 13 major prokaryotic phyla (99 bacteria and one archaeon), different lifestyles (e.g. free living, commensal, obligate intracellular) and various genome sizes (from 913Kb to 9.2 Mb). Detailed information about the dataset is provided Table S1. The maximum likelihood tree and the core genome of each species were used to infer the simulation parameters: GC-content, transition/transversion ratio, uneven substitution rates across codon positions, branch-specific substitution rates and tree topology (see Methods). Recombination rates *ρ* were expressed relative to mutations rates following a Poisson process. For each simulation, *ρ* was maintained as constant relative to the substitution rate across the branches of the tree. The donor and recipient genomes were randomly chosen between branches of the tree that overlapped in time. Each recombinant fragment size was pulled from a geometric distribution of mean 100 bp and all the sites of the core genome had the same probability to recombine. Following this procedure, we simulated 100 core genome alignments with recombination rates varying from *ρ* = 0 to *ρ* = 10 (with a step of 0.1) for each species. Recombination rates *ρ/m* were then transformed into effective recombination rates *r/m* (see Methods), which represents the effective number of alleles exchanged by recombination relative to substitutions introduced by mutations. Note that we generated the simulation parameters with a range of *ρ* values varying from 0 to 10 and a fixed value of *δ* in order to simulate genome evolution with an increasing range of *r/m* values in line with *r/m* values reported across species and studies [14, 41]. For each simulation, the phylogenetic tree was built from each simulated core genome, and the topologies of the simulated trees were compared to the true topology of the tree. We defined the tree topology score (TTS) as the percent of internal monophyletic clades shared by both trees (i.e. the percent of internal nodes that are composed of the same sets of strains). Average *r/m* and TTS values across the 100 species are presented Fig. 2; all *r/m* and TTS combined values across the 100 species are presented Figure S1A and *r/m* and TTS values are presented individually for each species Figure S2). Results are also presented for recombination rates expressed as *ρ/m* in Figure S3.

Our results revealed that while many trees are substantially impacted by recombination rates, the phylogenetic signal is not completely lost (Fig. 2). Tree topologies sharply declined for *r/m* values between 0 and 2; however, even high rates of recombination did not completely erode the phylogenetic signal of the core genomes. Indeed, the average tree topology score plateaus around 50% topology similarity even for high rates of recombination (average *r/m* = 3). This conservation of tree topologies was much higher than expected by chance (TTS =3% for comparisons against randomized tree topologies), indicating that even high levels of recombination are not completely obliterating the phylogenetic signal in these sequences. However, due to the variation of polymorphisms across species, several species presented much higher effective recombination rates (*r/m*) than others (i.e. more alleles were exchanged for the same number of recombination events) and those species with very high recombination rates (*r/m* > 7) yielded trees that were completely inconsistent with the true phylogeny (TTS = 0%, Figures S1A and S2). Note that simulations with high recombination rates (*r/m* > 2) represent cases where alleles are, on average, exchanged multiple times. In contrast to our results, a previous study found that core genome phylogenies were extremely robust to recombination [33], but this discrepancy likely stands from the fact that the cited analysis was conducted under more modest recombination rates compared to our study.

### Impact of recombination rate on bootstrap supports

**Fig. 3:**
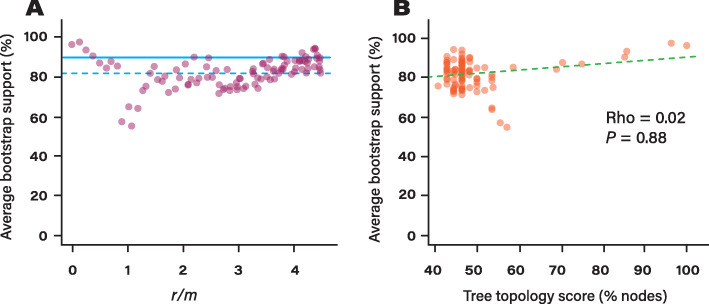
Impact of recombination rate on bootstrap supports. **a** Average bootstrap supports of the trees inferred with the core genomes of *A. pittii* simulated with different recombination rates (*r/m*). The trees and their bootstrap supports were inferred with RAxML. The blue dashed line represents the average bootstrap supports estimated across all the simulated trees of *A. pittii*. The blue solid line represents the average bootstrap support of the real tree of *A. pittii*. **b** Relationship between the average bootstrap supports of the trees of *A. pittii* simulated with various recombination rates and their tree topology score relative to the real tree of *A. pittii*

We tested to what extent recombination rates impact the bootstrap supports of the simulated trees. To do so, we computed the bootstrap supports of the maximum likelihood trees for the simulated core genomes of *A. pittii*. We observed a rather complex relationship between average bootstrap supports and recombination rates (Figs. 3a and S4), but we found an average bootstrap support of 82 (over 100) across all the simulated trees. Interestingly, average bootstrap supports were the highest for the trees simulated with either low recombination rates (*r/m* < 1) or high recombination rates (*r/m* > 3). Many of the simulated trees presented average bootstrap supports higher than the average bootstrap support observed in the real tree (89, Fig. 3a, blue solid line). In addition, we did not observe a significant correlation between the average bootstrap support of the simulated trees and their topology score relative to the real tree (Fig. 3b, Spearman’s Rho = 0.02, *P* = 0.88). These results indicate that recombination can result in incorrect trees presenting high bootstrap supports and that bootstrap supports therefore provide little information about the accuracy of the tree. In light of these observations, we recommend that the interpretation of bootstrap supports should be taken with care when assessing the quality of core genome phylogenies.

### Impact of phylogenetic methods on core genome phylogenies

**Fig. 4:**
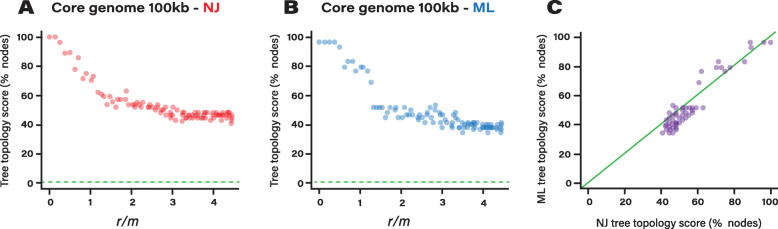
Impact of phylogenetic method on tree topology inference from core genome simulated with different levels of recombination. **a** Tree topology scores relative to recombination rates from trees inferred with RAxML. **b** Tree topology scores relative to recombination rates from trees inferred with BIONJ. **c** Relative performance of both phylogenetic approaches. Green line represents identical tree topology scores

We further tested which phylogenetic methods were the most adapted to reconstruct core genome phylogenies in the presence of recombination. We asked whether core genome phylogenies are best inferred by maximum likelihood (ML) methods or distance approaches using RAxML and BIONJ, respectively. We simulated core genome evolution using parameters of *A. pittii* under various recombination rates. Each simulated dataset was then used to reconstruct a ML and a distance-based phylogenetic tree. Each tree topology was then compared to the topology of the real tree and the tree topology score was computed as the number of shared nodes between the trees (Fig. 4a-b). Tree topology scores were found highly correlated, indicating that both methods yielded trees of overall similar quality. However, we observed that ML trees performed slightly but significantly better than distance trees, (*P* < 0.003, Wilcoxon signed-rank test, Fig. 4c). The better performance of ML trees was only observed when recombination rate was relatively low (*r/m* < 1).

### Impact of tree resolution on simulations

**Fig. 5:**
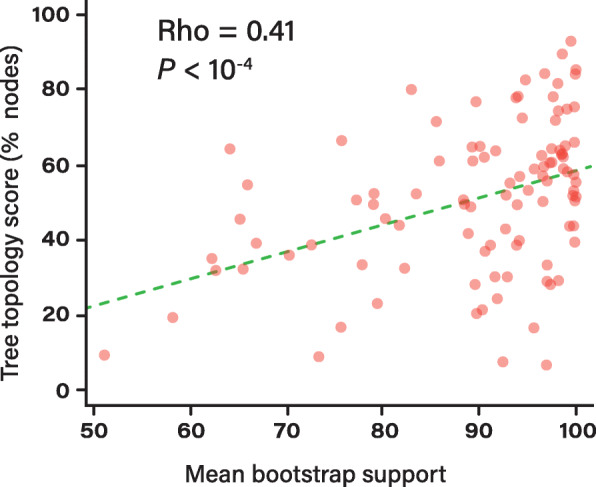
Correlation between average bootstrap values of the real tree and the average tree topology score. Bootstrap supports were inferred for the real tree of each species. The *y*-axis represents the average percent of nodes identical between the real tree and the simulated trees evolved with different recombination rates (i.e. each dot represents the average % of topology scores across the trees simulated with the different recombination rates for each species)

Our simulations produced a wide range of trees with various levels of topological similarities relative to the real trees, i.e. some datasets simulated with high recombination rates presented very similar topologies to the real trees, whereas others yielded inconsistent trees. We first hypothesized that these disparities were due to different levels of resolution across our dataset of real trees, since it is expected that poorly resolved trees are less likely to match the topology of the simulated trees. As expected, we found a strong positive correlation between the overall resolution of real trees (defined as the average bootstrap supports of all internal nodes) relative to the average topology score computed across all simulations (Fig. 5), revealing that the real trees that were originally poorly supported (low average bootstrap support), were most strongly affected by recombination. This indicates that many of the simulated trees were sensitive to recombination because the real tree was not robustly resolved.

Many simulated core genomes yielded inaccurate trees due to the poor resolution of the original tree; however, other core genomes yielded simulated trees that were strongly affected by recombination, although the original trees presented high bootstrap supports (average ≥ 90, Figures S1B and S2). In contrast, some core genomes produced well-resolved trees that matched the topologies of their simulated data even when exposed to high levels of recombination. This observation indicates that highly recombining species can yield inaccurate trees with high bootstrap supports. Our results indicate that even for well-supported trees (average bootstrap ≥90), less than 50% of the nodes are correctly inferred for *r/m* > 1.5. However, the robustness of a tree to recombination rate varied a lot across our dataset of species with well-supported trees (Figure S2), suggesting that other factors are influencing the robustness of trees to recombination rate.

### Recombination disrupts tree topology when selection is strong

**Fig. 6:**
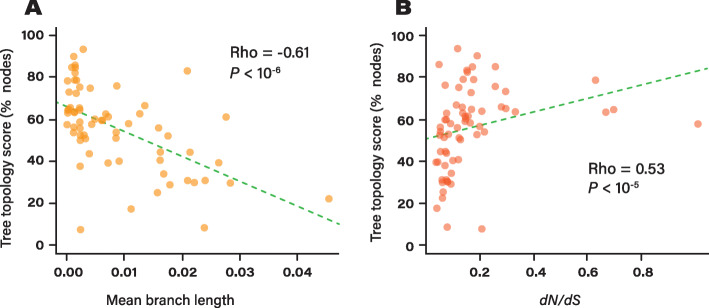
Impact of average branch length and selective pressures on tree robustness to recombination. **a** Correlation between average branch lengths of the tree and tree topology scores. **b** Correlation between average genomic *dN/dS* and tree topology scores

We further investigated the potential factors affecting the topology of the trees. From our set of trees with high average bootstrap supports (≥90, *n* = 65), we analyzed the impact of multiple parameters on tree quality. We observed that two parameters were significantly associated with the overall quality of the trees: the average branch length of the trees (Fig. 6. Spearman’s *Rho* = − 0.61, *P* < 10^− 6^) and the selective constraints as estimated by *dN/dS* ratios (Spearman’s *Rho* = 0.53, *P* < 10^− 5^). Note that these two parameters are not independent from one another since *dN/dS* is a time-dependent metric and more divergent genomes are known to present lower *dN/dS* estimates [42]. Nevertheless, these results suggest that, typically, more divergent core genomes and/or species experiencing stronger purifying selection are more likely to yield incorrect trees when affected by substantial levels of recombination. In theory, because the *r/m* metric of recombination rate is expressed relative to the substitution rates along the branches of the trees, the impact of recombination on tree topology should be independent from the overall branch length of the trees. For this reason, we hypothesize that differential selective pressures are most likely to be responsible for the uneven robustness of core genome phylogenies to recombination. We showed above that recombination does not impact the tree topology even when each site has been recombined randomly (Fig. 1). This could be explained by the fact that, under weaker selective pressures, alleles are more likely to be randomly exchanged across strains. In contrast, the species evolving under stronger selective pressures likely undergo more biased exchanges of alleles (i.e. though epistasis and selection against allele incompatibilities). Other parameters such as GC-content and the number of taxa in each tree were not significantly associated with tree robustness (Figure S5).

### Estimating the overall accuracy of core genome phylogenies

The range of recombination rates used in our simulation is higher than what most studies have reported for prokaryotes [14, 43]. It is difficult to give an overall estimate of recombination rate in prokaryotes, since these values are species-specific and different methodologies often give inconsistent estimates. Nevertheless, many species have been estimated to recombine with a rate ranging from 0 to 3, with a majority of species around *r/m* = 1 [14]. If we consider that a typical prokaryote species recombines with *r/m* = 1, we can anticipate that ~ 70% of the nodes of the reconstructed trees match the real tree topology. Under these approximate numbers, this would indicate that core genome phylogenies are generally correct for most prokaryote species, albeit not completely accurate.

### Caveats and limitations

Modelling homologous recombination in prokaryotes is a complex endeavor and reported recombination rates can vary greatly across datasets and methods [43]. We simulated the evolution of prokaryotic core genomes under a various set of recombination rates, but it remains difficult to estimate what constitutes typical recombination rates in these organisms. In contrast to a previous study [33], our set of parameters covered a broader range of recombination rates, which likely explains the difference in our conclusions. The recombination rates that we simulated appear more in line with rates typically reported in prokaryotes [14], but our analyses suggest that such results, inferred with phylogeny-based methods, are unlikely to yield accurate estimates when recombination rate is high and selection is strong. By using an empirical set of core genome phylogenies from prokaryote species encompassing diverse phyla, genome sizes and lifestyles, we attempted to simulate core genome evolution with recombination under more realistic conditions. However, recombination processes might be more complex in natural conditions, since rates of recombination can change over time and are likely impacted by population structure and ecology. Overall, more effort is needed to fully understand the relationship between recombination rate and population structure.

## Conclusion

Overall, this study revealed that core genome phylogenies can be very robust to high levels of recombination. Our results suggest that well-resolved trees—as inferred by bootstrap supports—do not necessarily represent accurate phylogenies. We conclude that recombination is much less likely to impact tree topologies when recombination events are random, as expected under weak selective pressures. Tree reconstruction is most strongly impacted by recombination for species evolving under strong selective regimes, presumably because higher selective pressures result in more biased exchanges of DNA [39, 44]. It is current practice to use highly conserved genes that evolve under strong purifying selection for phylogenetic analysis, since orthologous copies of these genes can be identified confidently. Although these genes likely represent the best gene markers for deep phylogenies, our results open the possibility that gene markers evolving under more relaxed selection might constitute better phylogenetic markers to reconstruct short-scale (i.e. strain-level) phylogenies when recombination rate is high. When recombination rates are very high (e.g. *r/m>* > 3) and selection is high, phylogenetic approaches are unlikely to infer meaningful trees and matrices of genomic distances might be more adequate objects than phylogenetic trees to represent relationships across genomes.

## Methods

### Datasets

We selected 100 core genome concatenates that were previously published [38] and constructed from genomes downloaded from the RefSeq database (ftp.ncbi.nlm.nih.gov/genomes/). The datasets with the least number of strains (13 ≤ *n* ≤ 55) were chosen for the analysis because complex datasets are much more computationally expensive to conduct the simulations and reconstruct phylogenetic trees. For each of these datasets, *dN/dS* ratios, normalized for divergence rate, were previously inferred [38]. Summary of the core genomes used in this study is given Table S1. The core genome concatenates are available in Dataset S1 and can be freely downloaded from https://www.kaggle.com/louismariebobay/core-genomes.

### Phylogenies

For each of the 100 datasets in this study, we reconstructed the phylogenetic tree with a maximum likelihood approach using RAxML v8 [45] under a Gamma + GTR (General Time Reversible) model [46]. For each core genome alignment, 100 true bootstrap replicates were generated using the same program and the same parameters. Phylogenetic trees built on simulated sequences were also built with RAxML v8 using the same parameters. The phylogenetic trees are available in Dataset S2 in Newick format and can be freely downloaded from https://www.kaggle.com/louismariebobay/core-genome. In addition, each simulated alignment was used to build a phylogenetic tree using a distance method with BIONJ [47].

### Simulations

We conducted independent sets of simulations for each of the 100 core genome datasets using *CoreSimul* [40]. Simulations were initiated by generating a random genome composed of 100,000 nucleotides with the same nucleotide composition (i.e. GC-content) of the real core genome for each species. The generated alignment was then evolved in silico with a branching process following the core genome tree of each species. The number of substitutions *m* was inferred from each branch of the real tree, and, for each branch of the simulation, the number of substitutions introduced in the simulated alignment was determined from a Poisson distribution of mean *m*. The spectrum of substitutions was set with a transition/transversion ratio *kappa* estimated from the real core genome of each species. In addition, the different positions across codons were evolved with different relative substitution rates, while maintaining the overall substitution rate estimated from each branch length of the tree. The relative substitution rates of each codon position were estimated based on the levels of polymorphisms observed at each codon position that were observed in the real core genome of each species. For each species, independent simulations were run using different recombination rates *ρ* ranging from *ρ* = 0 to *ρ* = 10, with a step of 0.1 (a total of 100 simulations per species). *ρ* is expressed as the number of recombination events relative to the number of substitutions *m* introduced by mutations. For each branch of the tree, the number of recombination events is defined from a Poisson distribution of mean *ρ.m*. Recombination tracts are chosen randomly in the sequence and the length of the recombination tract *δ* is randomly pulled from a geometric distribution of mean *δ* = 100 bp. This parameter was set to 100 bp because the average length of recombinant fragments is typically estimated to vary between 50 bp to 500 bp across studies and species [12, 48–51]. We used a fixed value of *δ* since we were interested in measuring the impact of recombination with the effective recombination rate *r/m* rather than the absolute recombination rate (see below). For each recombination event, the recipient genome is chosen randomly from the set of genomes overlapping in time (i.e. between contemporary branches of the tree). Mutations and recombination events are introduced in a randomized order for all branches of the tree overlapping in time. Concretely, all the branches of the tree are divided into segments of branches overlapping in time. The genomes of all overlapping segments are evolved simultaneously by introducing mutations and recombination events randomly. During the simulations we monitor the number of polymorphisms exchanged within each recombinant tract. We then define the effective recombination rate *r/m* as the number of polymorphisms *r* exchanged by recombination relative to substitutions introduced by mutations *m* so that *r/m* = *ρ.δ.ν* as in [52] where *ν* represents the average level of polymorphisms per recombination tract. The effective rate of recombination *r/m* is more frequently used to quantify recombination than the absolute rate of recombination defined by the parameters *ρ* and *δ*. Because *r/m* measures the number of alleles exchanged by recombination, it is a more informative metric to quantify the actual impact of recombination on genome evolution*.* Our simulations were therefore conducted with different values of *ρ* and a fixed value of *δ* in order to simulate genome evolution with an increasing range of *r/m* values similar to *r/m* values reported in previous studies [14, 41]. The simulated genomes were then used to build phylogenetic trees (see above) and the topology of each of these trees was compared to the topology of the tree built on the actual core genome of the corresponding species. The scripts used to run these simulations have been assembled into the *CoreSimul* program, which can be freely downloaded at https://github.com/lbobay/CoreSimul [40].

## Supplementary Information

**Additional file 1: Figure S1.** Impact of recombination rate (*r/m*) on tree inference across our set of 100 species. We evolved the core genome of 100 species to various levels of recombination rates. The tree topology score (TTS) represents the % of identical nodes between the simulated trees and the real trees. Horizontal green lines represent the average (plain line) percent and standard deviation (dashed lines) of nodes expected to be identical if the trees were random. **A** represents the relationship between *r/m* and TTS for all 100 species. **B** represents the relationship between *r/m* and TTS for the subset of species (*n* = 65) with well-resolved phylogenetic trees (average bootstrap support > 90 across all nodes).

**Additional file 2: Figures S2. I**mpact of recombination rate (*r/m*) on tree inference for each species tree. We evolved the core genome of 100 species to various levels of recombination rates. The tree topology score (TTS) represents the % of identical nodes between the simulated trees and the real trees. For each species, the average bootstrap value of the true phylogeny and the number of genomes (*n*) are indicated on top.

**Additional file 3: Figure S3.** Impact of recombination rate (*ρ/m*) on tree inference. We evolved the core genome of 100 species to various levels of recombination rates. The tree topology score (TTS) represents the % of identical nodes between the simulated trees and the real trees. Grey areas represent the standard deviation across the 100 species. Horizontal green lines represent the average (plain line) percent and standard deviation (dashed lines) of identical nodes expected to be identical if the trees were random.

**Additional file 4: Figure S4.** Impact of recombination rate (*ρ/m*) on bootstrap supports. **A** Average bootstrap supports of the trees inferred with the core genomes of *A. pittii* simulated with different recombination rates (*ρ/m*). The trees and their bootstrap supports were inferred with RAxML. The blue dashed line represents the average bootstrap supports estimated across all the simulated trees of *A. pittii*. The blue solid line represents the average bootstrap support of the real tree of *A. pittii*.

**Additional file 5: Figure S5.** Impact of genome numbers and GC-content on tree robustness to recombination. **A** Correlation between number of strains and tree topology scores. **B** Correlation between GC-content and tree topology scores.

**Additional file 6: Table S1.** Summary of the core genomes.

## Data Availability

The scripts used for this analysis are freely accessible on https://github.com/lbobay/CoreSimul. The datasets can be freely downloaded from https://www.kaggle.com/louismariebobay/core-genomes.
