# Supplementary figures and images for: Impact of homologous recombination on core genome phylogenies
Source: BMC Genomics. 2020 Nov 25;21:829. doi: 10.1186/s12864-020-07262-x (PMC7691112; doi:10.1186/s12864-020-07262-x)

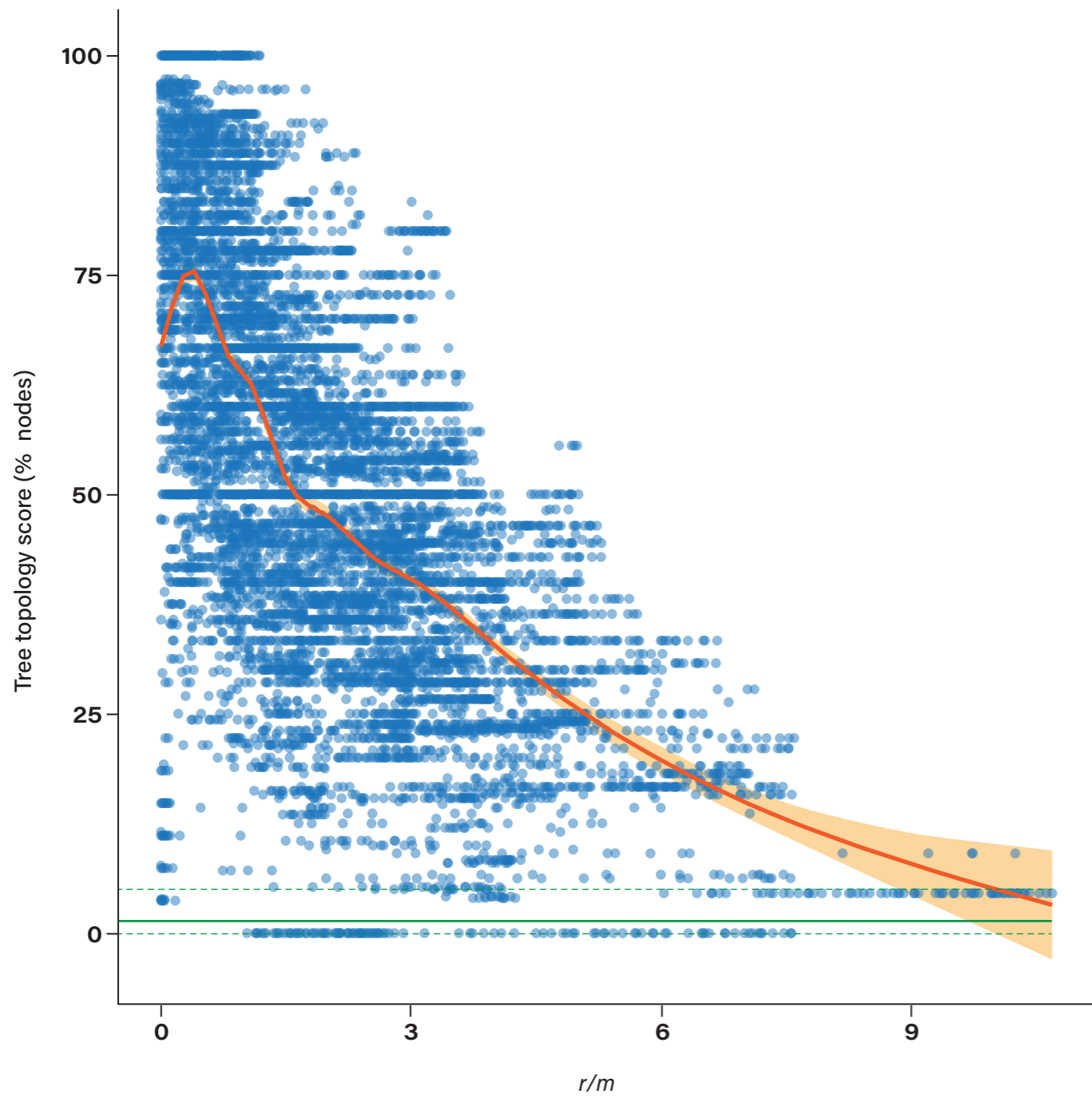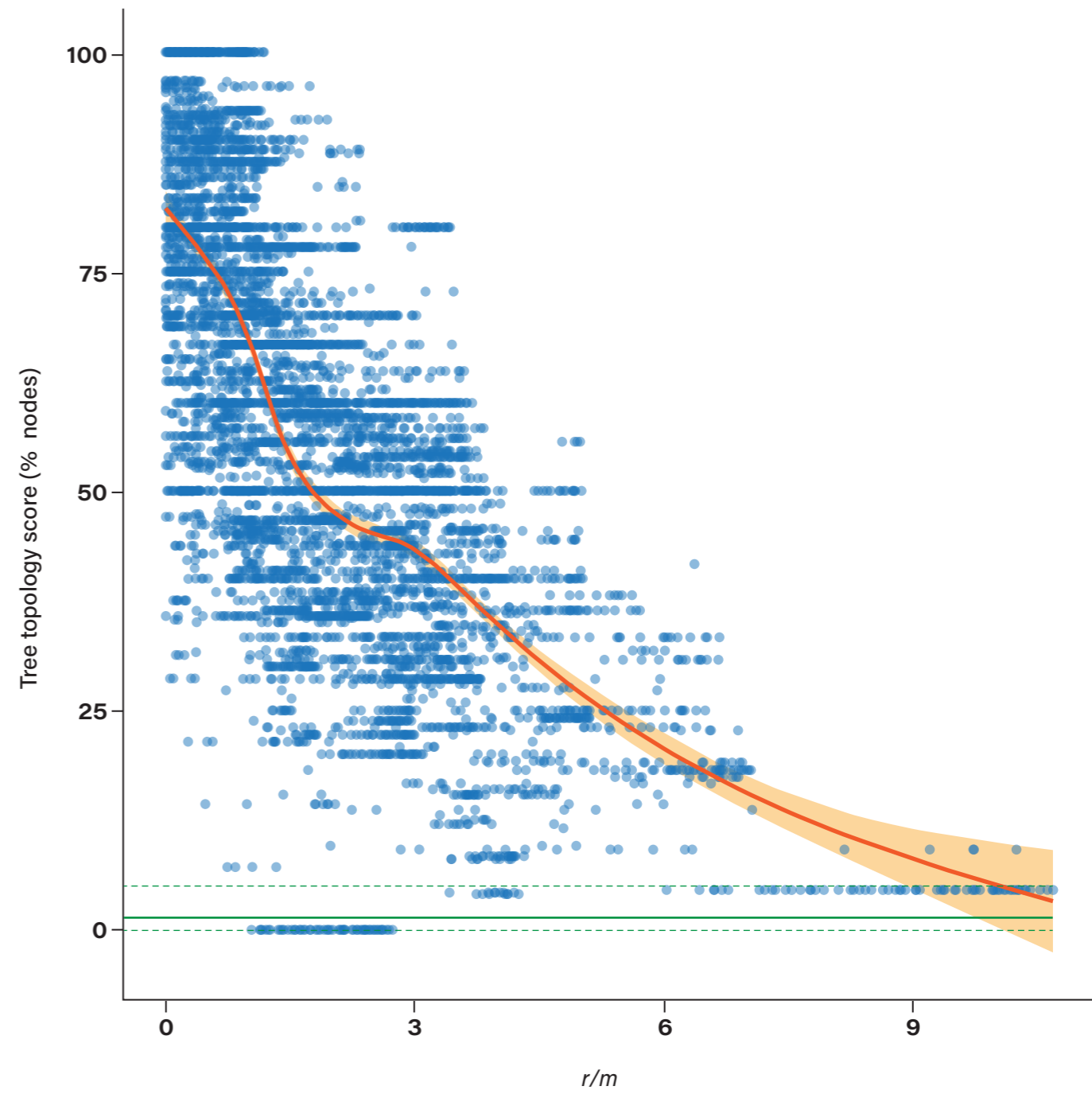

Supplement: Supplementary file 1 — Additional file 1: Figure S1. Impact of recombination rate (r/m) on tree inference across our set of 100 species. We evolved the core genome of 100 species to various levels of recombination rates. The tree topology score (TTS) represents the % of identical nodes between the simulated trees and the real trees. Horizontal green lines represent the average (plain line) percent and standard deviation (dashed lines) of nodes expected to be identical if the trees were random. A represents the relationship between r/m and TTS for all 100 species. B represents the relationship between r/m and TTS for the subset of species (n = 65) with well-resolved phylogenetic trees (average bootstrap support > 90 across all nodes). [file 12864_2020_7262_MOESM1_ESM.pdf]

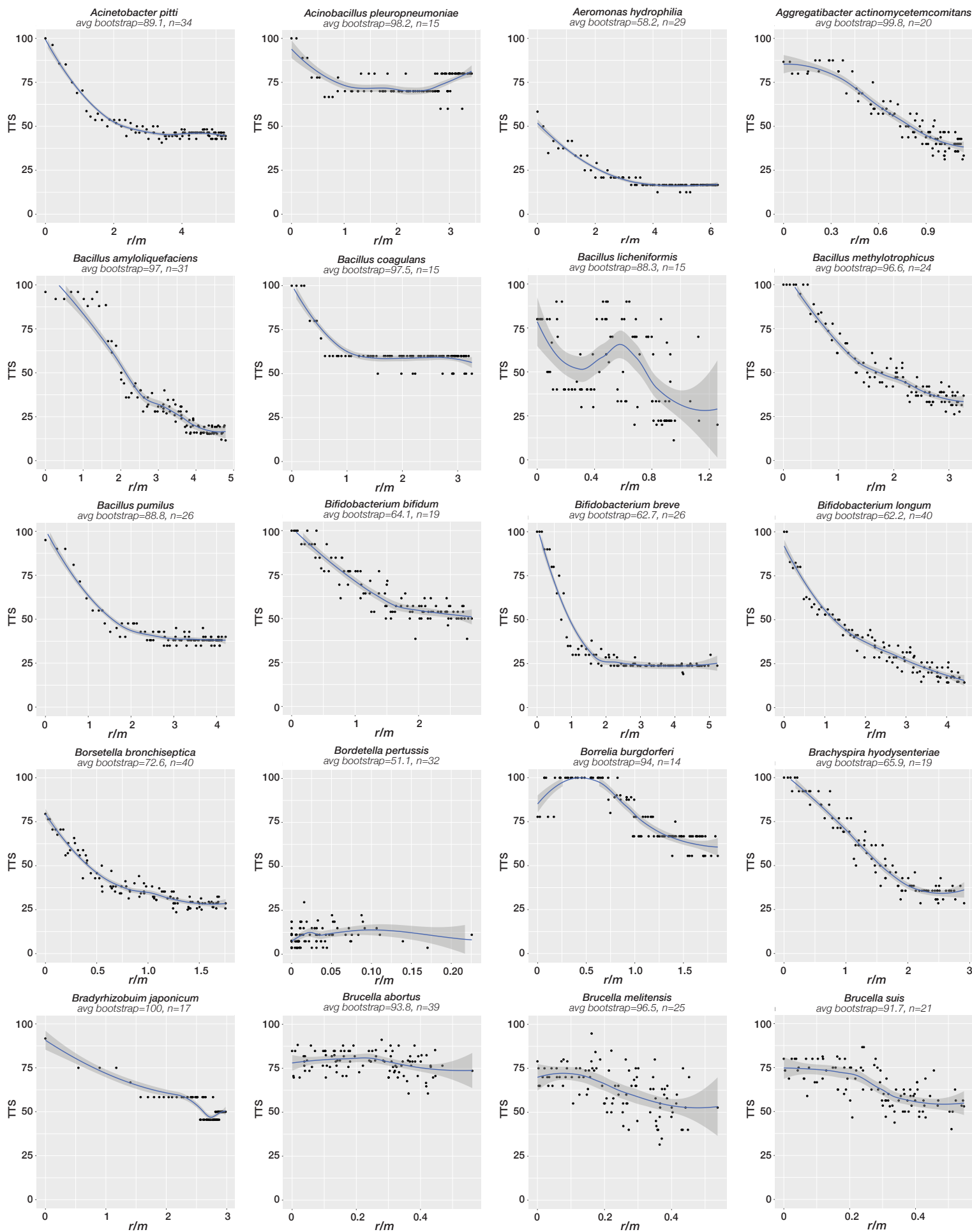

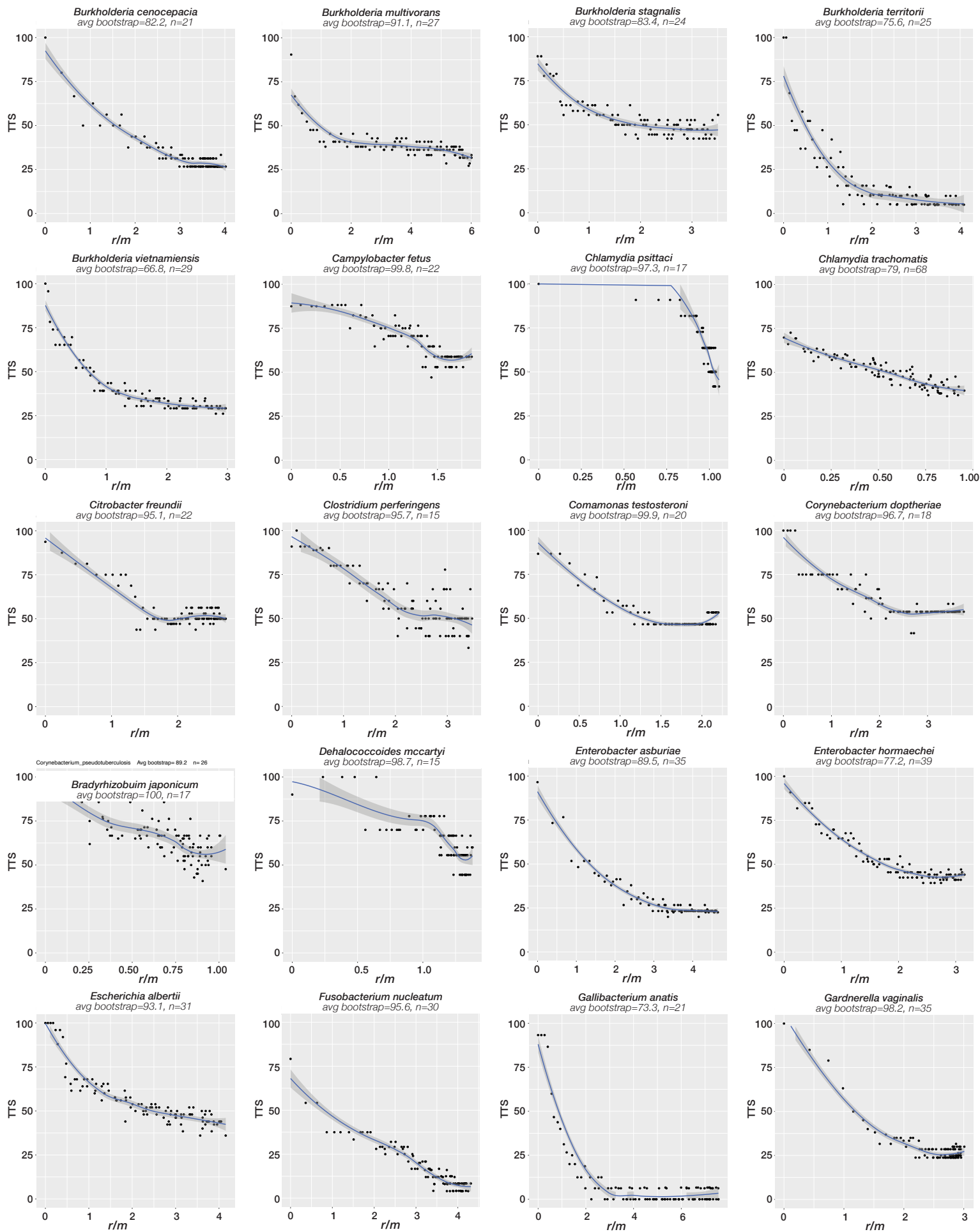

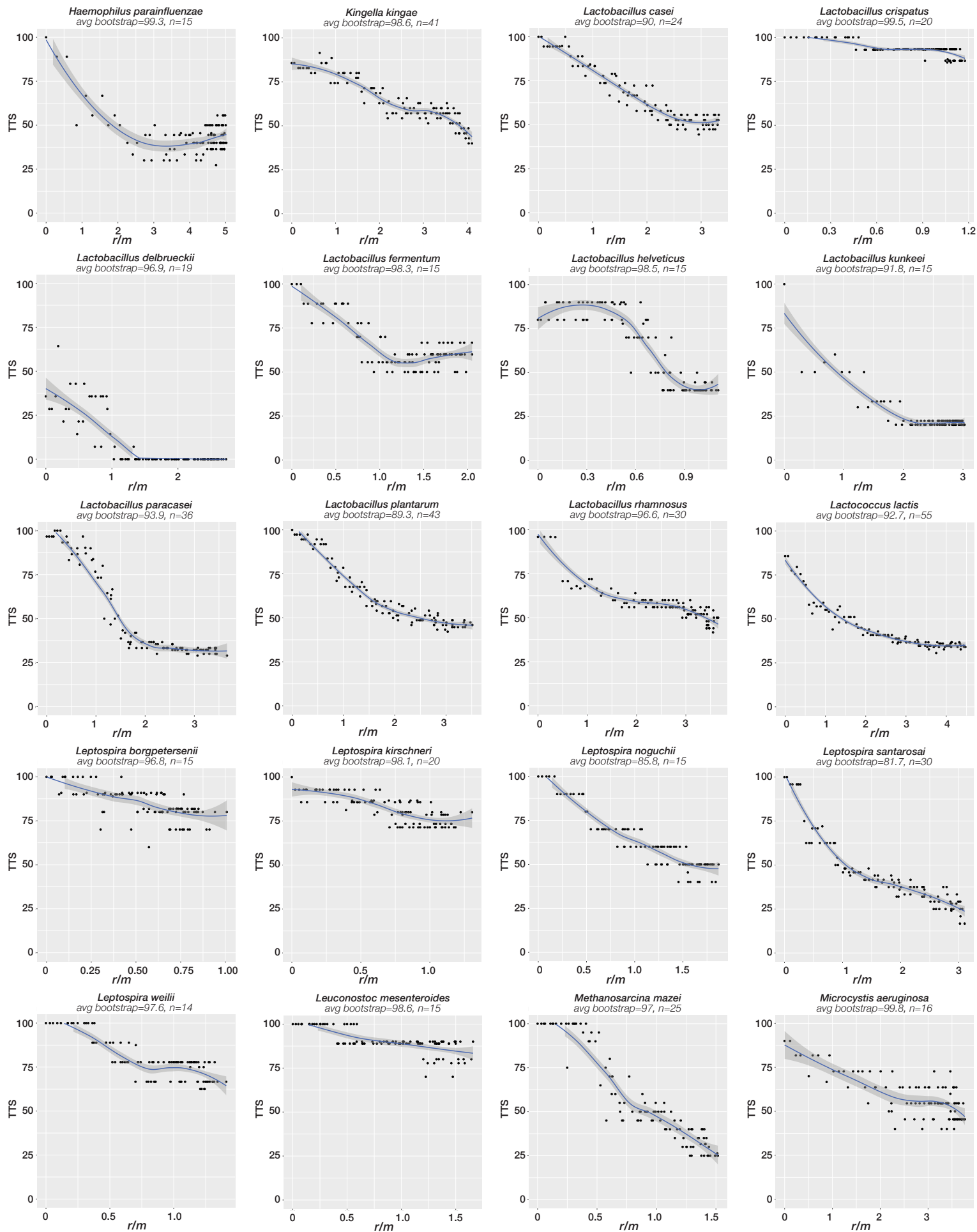

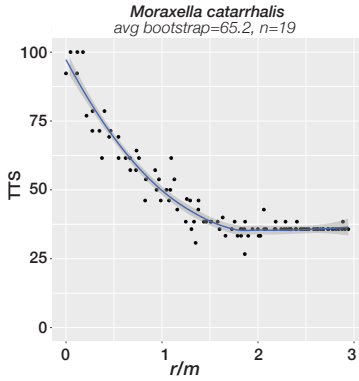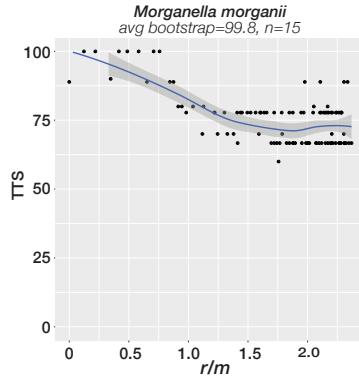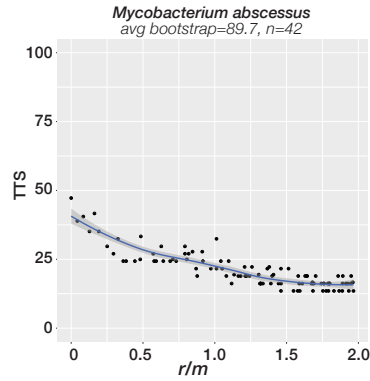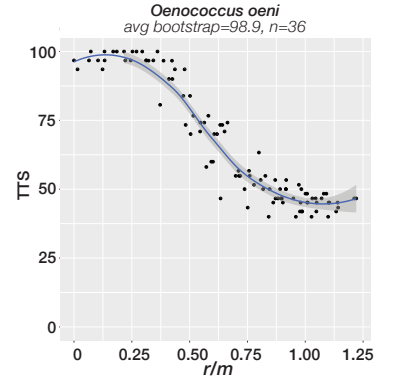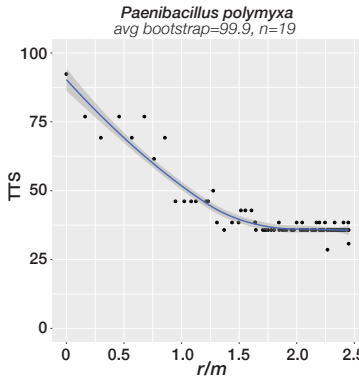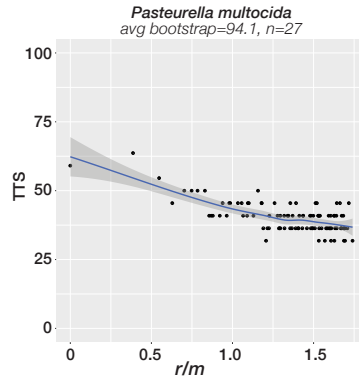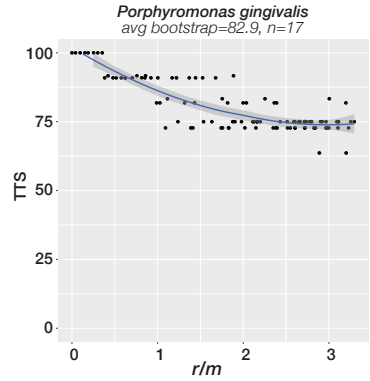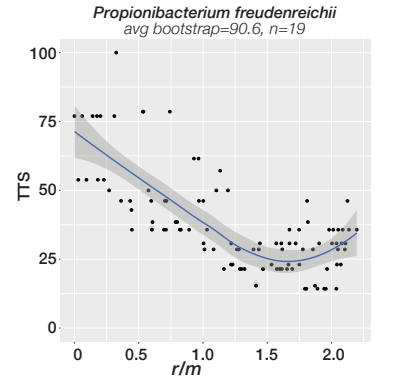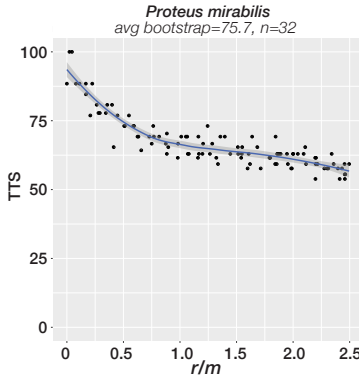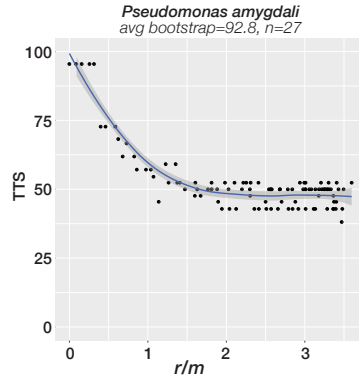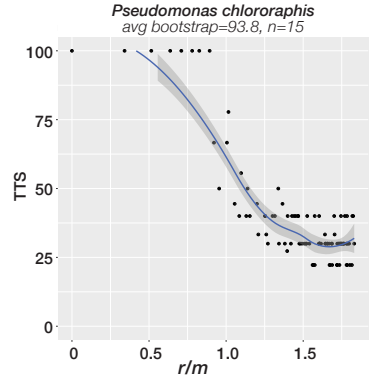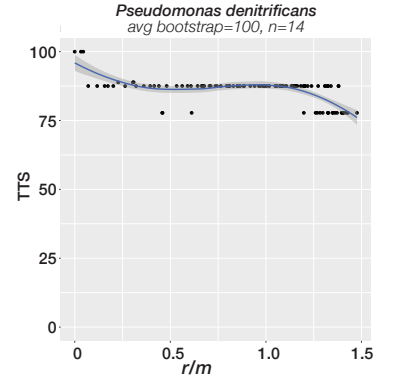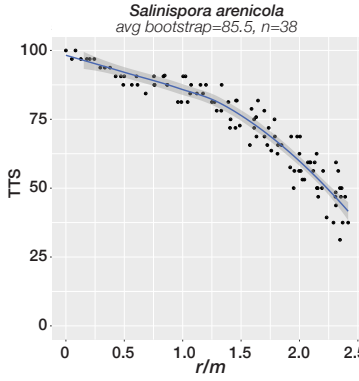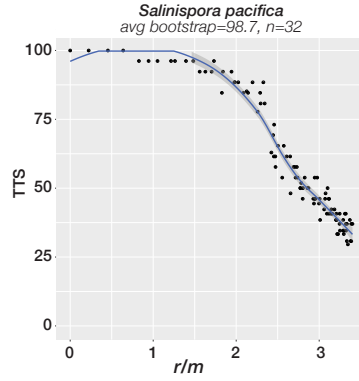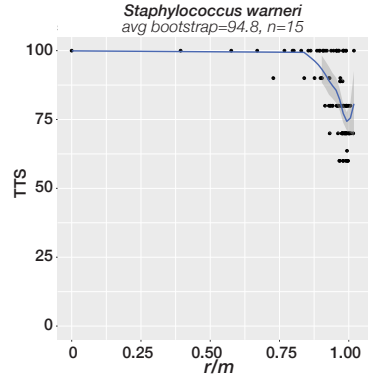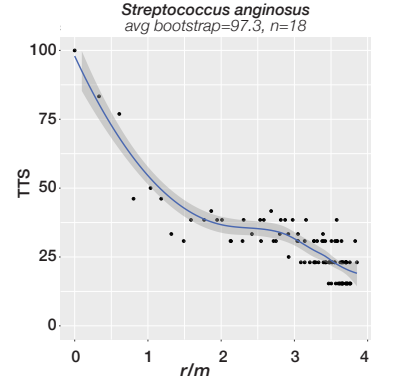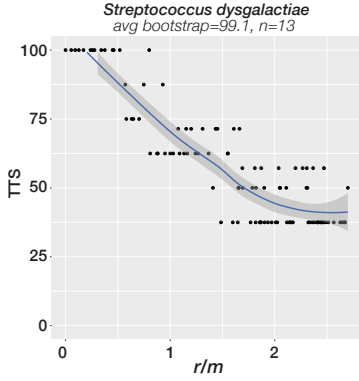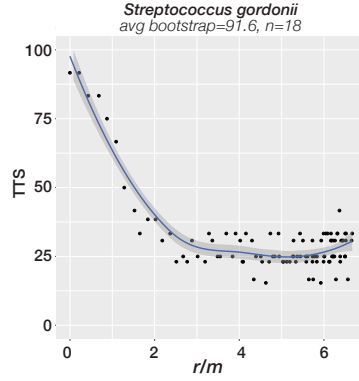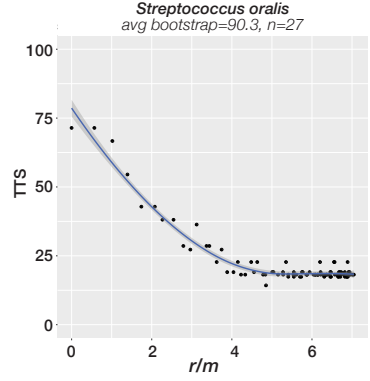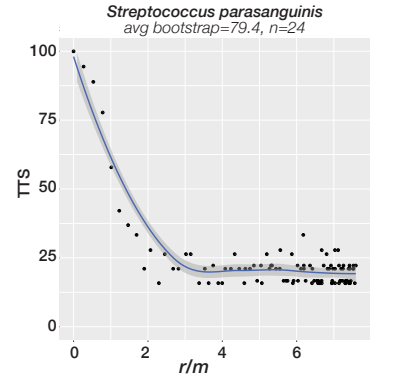

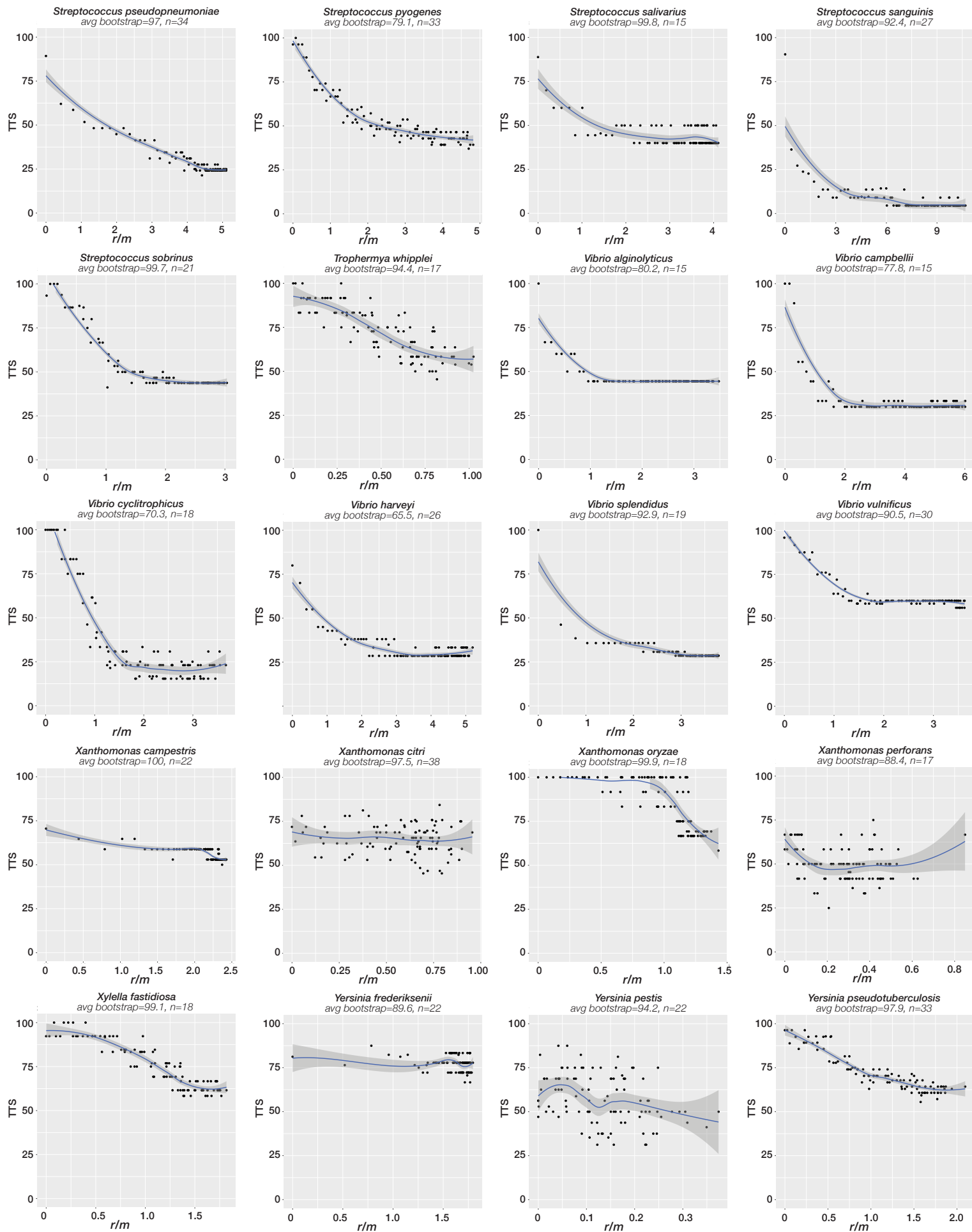

Supplement: Supplementary file 2 — Additional file 2: Figures S2. Impact of recombination rate (r/m) on tree inference for each species tree. We evolved the core genome of 100 species to various levels of recombination rates. The tree topology score (TTS) represents the % of identical nodes between the simulated trees and the real trees. For each species, the average bootstrap value of the true phylogeny and the number of genomes (n) are indicated on top. [file 12864_2020_7262_MOESM2_ESM.pdf]

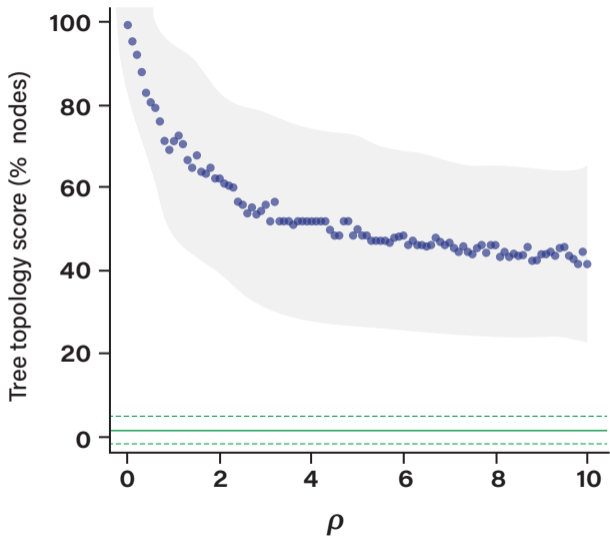

Supplement: Supplementary file 3 — Additional file 3: Figure S3. Impact of recombination rate (ρ/m) on tree inference. We evolved the core genome of 100 species to various levels of recombination rates. The tree topology score (TTS) represents the % of identical nodes between the simulated trees and the real trees. Grey areas represent the standard deviation across the 100 species. Horizontal green lines represent the average (plain line) percent and standard deviation (dashed lines) of identical nodes expected to be identical if the trees were random. [file 12864_2020_7262_MOESM3_ESM.pdf]

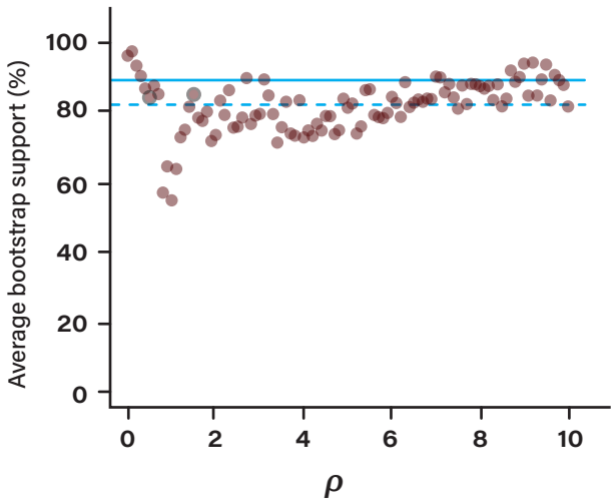

Supplement: Supplementary file 4 — Additional file 4: Figure S4. Impact of recombination rate (ρ/m) on bootstrap supports. A Average bootstrap supports of the trees inferred with the core genomes of A. pittii simulated with different recombination rates (ρ/m). The trees and their bootstrap supports were inferred with RAxML. The blue dashed line represents the average bootstrap supports estimated across all the simulated trees of A. pittii. The blue solid line represents the average bootstrap support of the real tree of A. pittii. [file 12864_2020_7262_MOESM4_ESM.pdf]

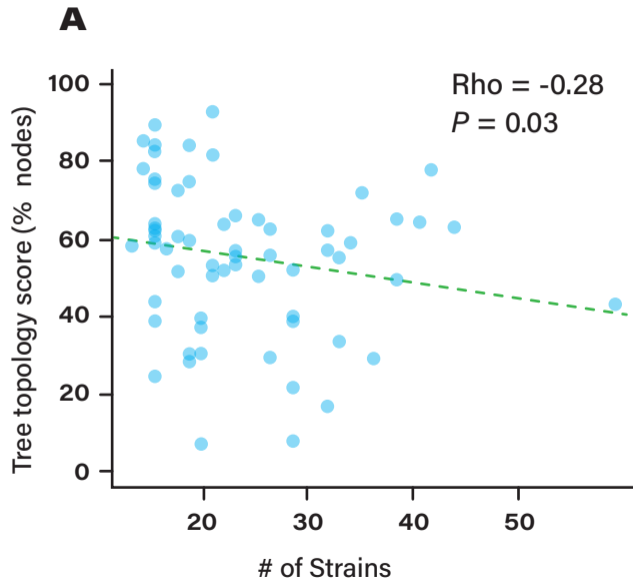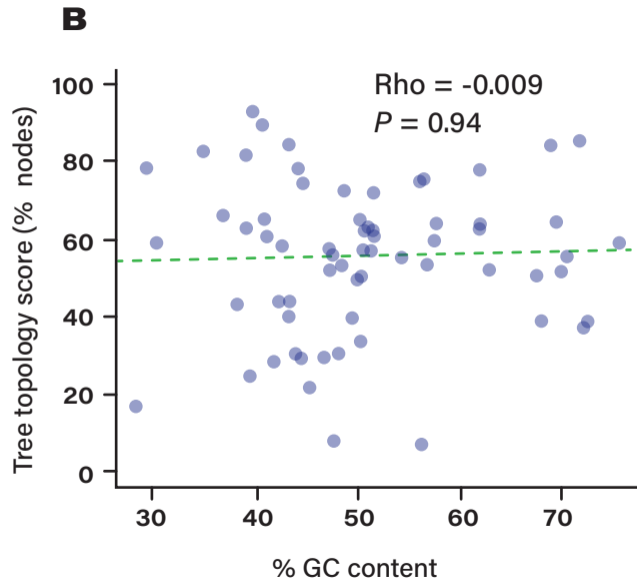

Supplement: Supplementary file 5 — Additional file 5: Figure S5. Impact of genome numbers and GC-content on tree robustness to recombination. A Correlation between number of strains and tree topology scores. B Correlation between GC-content and tree topology scores. [file 12864_2020_7262_MOESM5_ESM.pdf]
